# Studying the potential ameliorative effect of biosynthesized selenium nanoparticles using epigallocatechin gallate against depression in rats

**DOI:** 10.3389/fphar.2025.1691567

**Published:** 2026-01-13

**Authors:** Khaled M. Alam-ElDein, Ahmed H. I. Faraag, Nabil A. El-Yamany, Ahmed E. Abdel Moneim, Mohamed S. Abdelfattah, Manal F. El-khadragy, Heba A. Elmasry

**Affiliations:** 1 Department of Zoology and Entomology, Faculty of Science, Helwan University, Cairo, Egypt; 2 Molecular Biology and Biotechnology Department, School of Biotechnology, Badr University in Cairo, Badr, Egypt; 3 Botany and Microbiology Department, Faculty of Science, Helwan University, Cairo, Egypt; 4 Unit of Scientific Research, Applied College, Qassim University, Riyadh, Saudi Arabia; 5 Chemistry Department, Faculty of Science, Helwan University, Cairo, Egypt; 6 Department of Biology, College of Science, Princess Nourah bint Abdulrahman University, Riyadh, Saudi Arabia

**Keywords:** antidepressant, BDNF, behavioral tests, biosynthesis, depression, EGCG, neuroinflammation, NF-κB

## Abstract

**Introduction:**

Major depressive disorder (MDD) is a complex neuropsychiatric disorder with multifactorial origins involving oxidative stress, neuroinflammation, neurotransmitter imbalance, and HPA axis dysfunction. Conventional treatments are often limited by side effects and suboptimal efficacy, confirming the need for alternative therapies. This study investigates the antidepressant-like and neuroprotective potential of selenium nanoparticles biosynthesized using epigallocatechin gallate (SeNPs-EGCG) in a rat model of depression induced by chronic mild stress.

**Methods:**

Six groups of seven rats each were used in a model of depression caused by chronic unpredictable mild stress (CUMS): control, depressed, depressed treated with escitalopram, epigallocatechin gallate (EGCG), sodium selenite (Na_2_SeO_3_), and biosynthesised selenium nanoparticles capped with EGCG (SeNPs-EGCG). For 21 days, oral treatments were given. The open field test (OFT) and sucrose preference test (SPT) were used to measure depression-like behaviour. Oxidative stress markers, antioxidant enzymes, inflammatory cytokines, apoptotic proteins, monoamine neurotransmitters, corticosterone, BDNF, GFAP, and histopathological alterations were examined in prefrontal cortex tissue and serum.

**Results:**

Behavioral assays demonstrated that SeNPs-EGCG significantly reversed depression-like behaviors, evidenced by increased sucrose preference and grooming frequency in the SeNPs-EGCG-treated group compared to the depressed group. Biochemically, SeNPs-EGCG restored antioxidant defense by increasing GSH, SOD, and CAT levels, while reducing lipid peroxidation to near-normal levels. Neuroinflammatory markers such as TNF-α, IL-1β, IL-8, and NF-κB were markedly downregulated in the SeNPs-EGCG group. Molecular results also showed a slowing down of proapoptotic signals (Bax and Caspase-3) and upregulation of anti-apoptotic Bcl-2 and neurotrophic factor BDNF. Importantly, SeNPs-EGCG modulated key monoamines, increasing serotonin and DA levels. Compared to both EGCG and sodium selenite controls, SeNPs-EGCG demonstrated superior efficacy, comparable to the standard antidepressant escitalopram.

**Conclusion:**

The results underscore the multi-targeted mechanism of SeNPs-EGCG and suggest its promising role as a novel nano-based therapeutic strategy for depression.

## 1 Introduction

Depression is a complex and debilitating neuropsychiatric disorder marked by persistent low mood, cognitive dysfunction, anhedonia, and behavioral disturbances (Cui et al., 2024). Over 300 million individuals are affected by depression, significantly increasing the risk of suicide and chronic illness. While traditional views of depression have centered on monoamine imbalances, recent studies indicate that its etiology is multifactorial, involving oxidative stress, inflammation, neuroendocrine dysregulation, and neuronal apoptosis (Fila et al., 2021; Khedr et al., 2025).

A central feature of depression is the dysregulation of monoaminergic neurotransmission. Reduced levels of serotonin (5-HT) and dopamine (DA) neurotransmitters critical for mood regulation, motivation, and reward is commonly observed in depressive disorders. Additionally, increased activity of monoamine oxidase (MAO), the enzyme responsible for degrading these neurotransmitters, further exacerbates their depletion and has been strongly linked to depressive symptomatology (Cui et al., 2024; Zhao et al., 2025).

Depression is also characterized by elevated oxidative stress, which contributes to neuronal dysfunction and cell death. Increased levels of lipid peroxidation (LPO), commonly assessed via malondialdehyde (MDA), and nitric oxide (NO) reflect heightened oxidative burden in the brain. Simultaneously, antioxidant defenses are often impaired, as evidenced by low levels of glutathione (Ban et al., 2023) and diminished activity of antioxidant enzymes, such as superoxide dismutase (Gonçalves et al., 2021), catalase (CAT), glutathione peroxidase (GPx), and glutathione reductase (GR) (Liang et al., 2022; Winter et al., 2023).

Neuroinflammation plays a pivotal role in the progression of depressive disorders. Elevated concentrations of pro-inflammatory cytokines, such as tumor necrosis factor-alpha (TNF-α), interleukin-1 beta (IL-1β), and interleukin-8 (IL-8), alongside activation of nuclear factor kappa B (NF-κB) (Nafez, 2010), have been reported in both clinical and experimental models of depression. These inflammatory mediators can alter neurotransmission, disrupt synaptic plasticity, and promote neurotoxicity (Zhao et al., 2025; Gandhi et al., 2024).

Programmed neuronal cell death, or apoptosis, is another key pathological hallmark. Depression is associated with increased expression of pro-apoptotic markers such as Bax and caspase-3 and decreased expression of the anti-apoptotic protein Bcl-2, contributing to hippocampal atrophy and impaired neural function (Cong et al., 2023).

The neuroendocrine axis, particularly the hypothalamic-pituitary-adrenal (HPA) axis, is also dysregulated in depression (Georgiou et al., 2025; Mansour et al., 2025). Elevated corticosterone levels, rodents’ equivalent of human cortisol, reflect stress exposure and are linked to impaired neurogenesis, oxidative damage, and neuronal shrinkage in limbic structures such as the hippocampus and prefrontal cortex (Bains and Abdijadid, 2024).

Further, structural and functional abnormalities in glial cells have been identified. Reactive astrogliosis, indicated by elevated glial fibrillary acidic protein (GFAP), is commonly found in depressive brains and contributes to neuroinflammatory cascades and impaired neurotrophic support (Taha et al., 2023).

Brain-derived neurotrophic factor (BDNF), a key modulator of neuronal growth, synaptic plasticity, and resilience, is often downregulated in depression (Hao et al., 2021). The severity of depressive symptoms and their restoration are hallmarks of effective antidepressant treatments (Fila et al., 2021; Taha et al., 2023). Due to the limitations of conventional antidepressant therapies, such as delayed onset, side effects, and treatment resistance, there is increasing interest in exploring alternative, multi-targeted treatments. Herbal medicine has emerged as a valuable source of bioactive compounds with antidepressant potential (Zhao et al., 2025; Author Anonymous, 2025). Numerous plant-derived agents, including flavonoids, alkaloids, terpenoids, and polyphenols, have demonstrated efficacy in preclinical and clinical models of depression through antioxidant, anti-inflammatory, and neuroprotective mechanisms. Herbal remedies offer the advantage of low toxicity and synergistic activity across multiple biological pathways involved in depression (Cardoso et al., 2020; Edrees et al., 2007).

EGCG has a broad spectrum of biological activities, such as powerful antioxidant and anti-inflammatory properties (Dahiya et al., 2024). As well as the ability to modulate neurotransmitter systems, downregulate oxidative stress, inhibit MAO activity, and elevate BDNF levels, all of which are relevant to the treatment of depression (Babaei et al., 2025). EGCG has the ability to cross the barrier that surrounds the brain, known as the blood-brain barrier, and exert a direct protective effect on neurons by scavenging free radicals, regulating pro-inflammatory signalling pathways, and preserving mitochondrial integrity (Mao et al., 2019).

Selenium, an essential trace element, plays a vital role in maintaining brain health. It functions as a cofactor for several enzymes (Ansari et al., 2024). Selenium deficiency has been associated with mood disorders, cognitive decline, and impaired immune responses. It shows neuroprotective effects in various models of neurodegeneration and stress-related disorders through its ability to restore redox balance, reduce inflammation, and inhibit neuronal apoptosis (Elfakharany et al., 2024).

To enhance selenium’s therapeutic profile, it can be formulated into selenium nanoparticles (SeNPs). These nano-sized particles offer improved bioavailability, cellular uptake, and reduced toxicity compared to inorganic or organic selenium forms (Al Omairi et al., 2022). SeNPs exert a potent antioxidant and anti-inflammatory role and have demonstrated promise in the treatment of central nervous system disorders. Importantly, the reducing as well as stabilizing capacity of EGCG makes it suitable for use in the biosynthesis of selenium nanoparticles; the resulting SeNPs-EGCG possess synergistic properties that combine the neuroprotective benefits of both selenium and EGCG (Al Omairi et al., 2022; Alrashdi et al., 2023). This nanoformulation has shown enhanced biological activity, including greater free radical scavenging, anti-apoptotic signaling, and neuronal protection compared to EGCG or SeNPs alone (Wang et al., 2022).

Although SeNPs and EGCG have each been studied for their antioxidant and neuroprotective effects, their combined application in depression has not been explored. The present work is the first to report the green synthesis of EGCG-functionalized SeNPs and to demonstrate their synergistic efficacy in attenuating oxidative stress, neuroinflammation, and behavioral deficits in a validated depression model. This strategy highlights a novel therapeutic avenue that extends beyond the effects of either compound alone, in modulating monoamine neurotransmitters, inhibiting MAO activity, reducing oxidative and inflammatory markers, normalizing corticosterone levels, restoring BDNF expression, attenuating apoptosis, and regulating glial activation. Therefore, we aimed to study the potential antidepressant effects of SeNPs-EGCG in a rat model of depression, evaluating a comprehensive panel of biochemical, molecular, and neuroendocrine parameters.

## 2 Materials and methods

### 2.1 Drugs

EGCG (EGCG; CAS No. 989-51-5) was procured from Sigma-Aldrich (St. Louis, MO, USA) and used without further modification. To biosynthesize EGCG-conjugated selenium nanoparticles (SeNPs-EGCG), 10 mL of aqueous sodium selenite (Na_2_SeO_3_, 10 mM) was combined with an equal volume of EGCG solution (3.5 mg/mL). The mixture was subjected to continuous magnetic stirring for 12 h at ambient temperature to facilitate nanoparticle formation through reduction and capping processes. The resulting colloidal solution exhibited a characteristic red hue, indicative of nanoparticle synthesis, and was subsequently lyophilized using a vacuum freeze-drying system (Labconco FreeZone 4.5 L, Marshall Scientific, Hampton, NH, USA). The dry SeNPs-EGCG powder obtained was used for downstream experimental applications.

Following synthesis, the SeNPs-EGCG were stored in solution at 4 °C in an amber-colored glass bottle to protect against light exposure and maintain stability. Before each use, the suspension was briefly sonicated (1–2 min) to ensure homogenous dispersion and prevent nanoparticle aggregation.

#### 2.1.1 Characterization of nanoparticles

##### 2.1.1.1 Zeta size and potential analysis

The physicochemical properties of the biosynthesized selenium nanoparticles (SeNPs) were evaluated using a Zeta sizer Nano ZS90 (Malvern P Analytical Ltd., UK) at Helwan University to determine size distribution and surface charge. Measurements were carried out in ultrapure water at 25 °C, with samples sonicated prior to analysis to minimize aggregation. Dynamic light scattering (DLS) was employed to assess hydrodynamic size, yielding a Z-average of 32.19 nm with a polydispersity index (PDI) of 0.494, indicating moderate uniformity (Kheirollahi-Kouhestani et al., 2009). Intensity distribution analysis revealed two main populations: 74.47 nm (74% intensity) and 13.28 nm (26% intensity) (data not shown). Zeta potential was determined via electrophoretic light scattering, showing a strongly negative value of −61.0 ± 8.37 mV, confirming excellent colloidal stability due to high electrostatic repulsion (data not shown). Conductivity of the dispersion was 0.413 mS/cm, and measurement quality was validated as good. These results confirm the successful synthesis of stable, nanosized SeNPs suitable for subsequent biological applications (Clogston and Patri, 2010).

##### 2.1.1.2 Transmission electron microscopy

The morphological characteristics and nanoscale architecture of the biosynthesized selenium nanoparticles (SeNPs) were examined using high-resolution transmission electron microscopy (TEM; JEOL Ltd., Mitaka, Tokyo, Japan) at Al-Azhar University. Prior to imaging, a small aliquot of the aqueous nanoparticle suspension was ultrasonicated to prevent aggregation and subsequently deposited onto a carbon-coated copper grid. Excess fluid was carefully removed with filter paper, and the grids were air-dried under ambient conditions before analysis (Rao et al., 2010). TEM micrographs were obtained at appropriate accelerating voltages, enabling high-contrast visualization of nanoparticle features at the nanometer scale. This technique provided direct confirmation of particle integrity, surface morphology, and spatial distribution (Van Erum et al., 2019). TEM analysis ensured a comprehensive understanding of SeNPs morphology, which is critical for correlating their physicochemical attributes with potential biological performance.

##### 2.1.1.3 UV–vis spectroscopy and stability evaluation

The optical characteristics of the biosynthesized SeNPs-EGCG were examined using a UV–Vis spectrophotometer (Shimadzu, Japan) in Badr University in Cairo, Egypt. Spectral scans were performed in the range of 200–800 nm to detect the surface plasmon resonance (SPR) band and verify nanoparticle formation and functionalization by EGCG. To assess colloidal stability, nanoparticle suspensions were stored at room temperature in the dark, and their absorption spectra were recorded at monthly intervals for a period of 6 months.

All SeNPs-EGCG used in this study originated from a single synthesized batch, which was fully characterized and aliquoted for storage. The same batch was used for all experimental groups to avoid batch-to-batch variability.

### 2.2 Experimental design

#### 2.2.1 Experimental animals

Healthy adult male albino rats (100–120 g) were used in this study. The animals were housed in polycarbonate cages fitted with stainless-steel wire tops, with a maximum of five rats per cage, and provided with wood shavings as bedding. They were maintained under a controlled environment with a 12:12 h light-dark cycle, an ambient temperature of 22 °C ± 3 °C, and regulated relative humidity. Standard laboratory chow and water were made available *ad libitum*. All animals were acclimatized to laboratory conditions for 2 weeks prior to the commencement of experimental procedures. Animal care and experimental protocols were conducted in accordance with the ethical guidelines for the use of laboratory animals and were approved by the Department of Zoology, Faculty of Science, Helwan University (Approval No. HU/z28-03-2024).

#### 2.2.2 Experimental grouping

The experiment involved 42 animals, divided into six groups, with 7 animals per group subjected to specific conditions and treatments. The animals were grouped as follows:

##### 2.2.2.1 Control group (CNT, n = 7)

Animals of this group will be exposed to standard conditions for 28 consecutive days. At day 7, animals of this group will receive normal saline solution (0.9% NaCl) for 21 consecutive days.

##### 2.2.2.2 Depressed group (DEP, n = 7)

Animals of this group will be subjected to a protocol of chronic unpredictable mild stress for 28 consecutive days. At day 7, animals of this group will receive normal saline solution (0.9% NaCl) for 21 consecutive days.

##### 2.2.2.3 Depression treated with standard drug group (DEP/SD, n = 7)

Animals of this group will be subjected to a protocol of CUMS for 28 consecutive days. On day 7, animals of this group will receive daily oral administration of the standard drug (escitalopram) for 21 consecutive days in a dose of 10 mg/kg/day (Bech et al., 2006).

##### 2.2.2.4 Depression treated with epigallocatechin gallate group (DEP/EGCG, n = 7)

Animals of this group will be subjected to a protocol of CUMS for 28 consecutive days. At day 7, animals of this group will receive daily oral administration of 50 mg/kg/day of EGCG for 21 consecutive days (Loftis et al., 2013).

##### 2.2.2.5 Depression treated with the sodium selenite group (DEP/Na_2_SeO_3_, n = 7)

Animals of this group will be subjected to a protocol of CUMS for 28 consecutive days. At day 7, animals of this group will receive daily oral administration of 0.5 mg/kg/day of Se nanoparticles for 21 consecutive days (Hezema et al., 2025; Lutfy et al., 2025).

##### 2.2.2.6 Depression treated with se nanoparticles biosynthesized using the epigallocatechin gallate group (DEP/SeNPs-EGCG, n = 7)

Animals of this group will be subjected to a protocol of CUMS for 28 consecutive days. On day 7, animals of this group will receive daily oral administration of 0.5 mg/kg/day of Se nanoparticles biosynthesized using EGCG for 21 consecutive days (Alrashdi et al., 2023; Lutfy et al., 2025).

### 2.3 Induction of depression via chronic unpredictable mild stress

To establish a depression-like phenotype, rats were subjected to the CUMS paradigm, a widely recognized and validated protocol for modeling depressive symptomatology in rodents. Over a 3-week period, animals were exposed to a repertoire of mild stressors delivered in a random and unpredictable sequence to minimize habituation. The stressors employed included cage tilting at 45 °C for 12 h, continuous overnight illumination, damp bedding (200 mL water mixed with sawdust), social isolation, forced swimming for 5 min in water maintained at 18 °C, intermittent food or water deprivation (12–24 h), and paired housing with unfamiliar conspecifics (Antoniuk et al., 2019).

Each stressor was applied no more than once every 48 h, and their timing and sequence were randomized daily. This protocol was designed to mimic the mild, unpredictable stress that contributes to the development of human depression. The cumulative impact of these stressors resulted in behavioral, biochemical, and neuroendocrine alterations consistent with depressive-like states, including anhedonia, locomotor suppression, oxidative imbalance, neuroinflammation, and imbalance of the HPA axis (Luo et al., 2025).

### 2.4 Behavioral assessments

To evaluate the behavioral phenotype associated with depression-like states, two validated paradigms were employed. The Open Field Test (OFT) was used to assess general locomotor activity and anxiety-related behavior. Animals were individually placed in the center of a Plexiglas arena, and parameters such as total distance traveled, time spent in the center, and rearing frequency were recorded (Walsh and Cummins, 1976; Gould et al., 2009).

Additionally, the Sucrose Preference Test (SPT) was conducted as a measure of anhedonia, which is a core symptom of depression. Following an adaptation period with two water bottles (one containing 1% sucrose solution), animals were presented with free access to both sucrose and water for 2 h. Sucrose preference was calculated as the volume (mL) of sucrose solution consumed relative to the total liquid intake (Markov, 2022).

### 2.5 Tissue collection and sample preparation

Upon completion of behavioral testing, animals were euthanized under deep anesthesia. The brain was rapidly excised, and the prefrontal cortex region was carefully dissected, rinsed with ice-cold physiological saline to ensure it was free of blood, and subdivided for various analyses.

For biochemical assays and corticosteroid hormone tests, blood was collected directly and centrifuged at 5,000 rpm for 10 min to obtain serum. The first portion of the prefrontal cortex was homogenized in 10 mM phosphate buffer solution (pH 7.4). This preparation was used for subsequent oxidative stress, antioxidant, inflammatory, apoptotic, and neurochemical assessments. For monoamine neurotransmitter analysis, an additional cortical portion was homogenized in 75% HPLC-grade methanol (10% w/v), then centrifuged at 4,500 rpm for 12 min. The resulting supernatant was collected and analyzed via high-performance liquid chromatography (HPLC). For histological and immunohistochemistry analysis, samples of the prefrontal cortex were stored in 10% neutral-buffered formalin for the fixation process, then the tissue was embedded in paraffin wax and sectioned for staining and microscopic evaluation.

### 2.6 Biochemical assays

All biochemical analyses were conducted on the prefrontal cortex homogenates except corticosterone, which was measured in serum using commercially available ELISA kits, in accordance with the manufacturers’ protocols.

#### 2.6.1 Assessment of oxidative and antioxidant status

Oxidative stress parameters included the quantification of LPO, assessed as MDA (Ohkawa, 1982), and NO levels measured at 540 nm (Green et al., 1982). Ellman’s method was used to analyze GSH levels as a marker of cellular redox status (Ellman et al., 1961). To evaluate the antioxidant defense system, the activities of SOD, CAT, GPx, and GR were determined using previously described techniques (Paglia and Valentine, 1967; Nishikimi et al., 1972).

#### 2.6.2 Inflammatory/anti-inflammatory marker evaluation

Inflammatory status in cortical tissue was evaluated by measuring the protein concentrations of key pro-inflammatory cytokines: tumor necrosis factor-alpha (TNF-α), interleukin-1 beta (IL-1β), and interleukin-8 (IL-8), using ELISA.

#### 2.6.3 Apoptotic/anti-apoptotic marker analysis

Apoptotic and anti-apoptotic markers were quantified using commercially available.

ELISA kits. Bax (Cat. No. MBS722913) and Caspase-3 (Cat. No. MBS701377) were measured using kits from MyBioSource (San Diego, CA, USA), whereas Bcl-2 was determined using a Cloud-Clone ELISA kit (Cat. No. SEB691Ra; Cloud-Clone Corp, Houston, TX, USA) All assays were performed according to the manufacturer’s instructions.

#### 2.6.4 Monoaminergic neurotransmitter quantification

Concentrations of cortical 5-HT and DA were determined from methanol extracts using HPLC equipped with an electrochemical detector (Pagel et al., 2000). This allowed for accurate profiling of monoaminergic dysregulation associated with depressive-like behaviors.

#### 2.6.5 Gene expression analysis

Gene expression was evaluated using quantitative reverse transcription polymerase chain reaction (RT-qPCR). Total RNA was extracted from prefrontal cortex tissue, and complementary DNA was synthesized. The relative mRNA expression levels of MAO and nuclear factor kappa B (NF-κB) were quantified using gene-specific primers. GAPDH served as the internal reference gene for normalization. Table 1. Gene expression levels were normalized to GAPDH as the internal housekeeping control, and relative quantification was performed using the 2^–ΔΔCt method. Primer specificity and efficiency (90%–110%) were validated through melt curve analysis and standard curve assessments, respectively. For each gene, biological replicates were obtained from all animals within each group, and each sample was run in technical triplicates to ensure accuracy and reproducibility.

**TABLE 1:** Primer sequences of genes analyzed by RT-qPCR.

Genes	Forward sequence	Reverse sequence	Accession number
*MAO*	TGC​ATG​GTG​TAT​TAC​AAG​GA	CTT​GAG​ATC​CCA​GAA​CTT​TG	NM_001270458.1
*NFκB*	GTC​TCA​AAC​CAA​ACA​GCC​TCA​C	CAG​TGT​CTT​CCT​CGA​CAT​GGA​T	NM_199267.2
*GAPDH*	ATG​GTG​AAG​GTC​GGT​GTG​AAC​G	TGG​TGA​AGA​CGC​CAG​TAG​ACT​C	NM_001411843.1

#### 2.6.6 Hormonal analysis

To assess HPA axis function, Serum corticosterone levels were assessed using the Biosensis Mature Corticosterone Rapid™ ELISA Kit (Cat. No. BEK-2227-1P; Biosensis Pty Ltd., Thebarton, Australia). All assays were performed according to the manufacturer’s instructions.”

#### 2.6.7 Assessment of glial reactivity and neurotrophic factor expression

To assess the glial activation in response to treatment, activity of glial fibrillary acidic protein (GFAP), a key marker of astrocytic reactivity, was quantified in prefrontal cortex tissue using a commercially available enzyme-linked immunosorbent assay (Winter et al., 2023) kit (Cat. No. NS830; Merck, Darmstadt, Germany), following the manufacturer’s validated protocol (Towers, 1998).

In parallel, brain-derived neurotrophic factor (BDNF), a pivotal mediator of synaptic plasticity and neuronal survival, was measured using an ELISA kit sourced from My Bio-Source (Cat. No. MBS355435; San Diego, CA, USA). Assays were performed according to the supplier’s technical instructions, ensuring optimal specificity and sensitivity.

### 2.7 Immunohistochemical analysis

To visualize astrocyte activation, paraffin-embedded cortical sections were immunostained for GFAP. Briefly, 4–5 µm sections were deparaffinized, rehydrated, and incubated with primary anti-GFAP antibodies, followed by appropriate biotinylated secondary antibodies and streptavidin-peroxidase complexes. Detection was achieved using 3,3′-diaminobenzidine (DAB) as a chromogen, and sections were counterstained with hematoxylin.

### 2.8 Quantitative assessment of IHC staining

This step is an effective tool for studying protein location inside tissue. Semi-quantitative IHC is performed utilizing ImageJ Fiji software, version 1.2 (without employment of specific plugin). Deconvolution and downstream analysis were performed. GFAP immunoreaction area percentage was assessed at magnification of X 400 for all groups.

### 2.9 Histopathological examination

Fixed prefrontal cortex tissue sections were stained with hematoxylin and eosin (H&E) for general histological evaluation. Neuronal morphology and integrity were examined under a light microscope to identify degenerative changes associated with depressive pathology or treatment response.

### 2.10 Statistical analysis

The data were analyzed and expressed as mean ± standard error (SE). They were analyzed by one-way analysis of variance (ANOVA) according to Tukey (1995). The statistical package (Graphpad Prism 9) was used to compare all the treated groups. The changes between the means were considered significant when the P < 0.05 (Kim, 2014).

## 3 Results

Following the comprehensive assessment of behavioral, biochemical, neurochemical, and endocrine parameters, three additional experimental groups were included to evaluate the safety profile of EGCG, Na_2_SeO_3_, and SeNPs-EGCG in non-stressed control animals. Across all measured outcomes, these compounds did not produce any significant deviations from baseline values observed in the untreated control group. This lack of adverse effect strongly suggests that EGCG, Na_2_SeO_3_, and SeNPs-EGCG are well-tolerated and exhibit no inherent toxicity under physiological conditions, supporting their safety for further therapeutic application.

### 3.1 SeNPs-EGCG characterization

#### 3.1.1 Transmission electron microscope imaging

**FIGURE 1:**
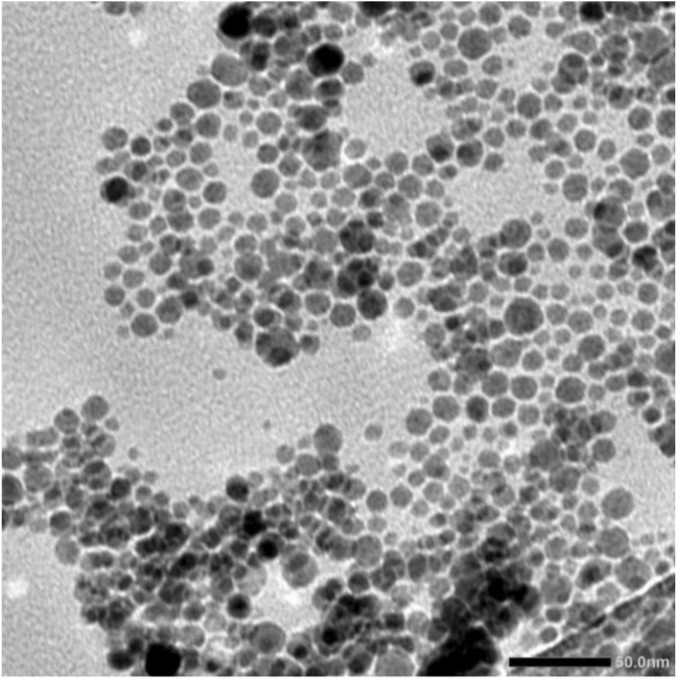
TEM of EGCG-functionalized selenium nanoparticles (SeNPs-EGCG) showing spherical particles with relatively uniform morphology and sizes mostly ranging between 20 and 50 nm.

The TEM image shows that the EGCG-mediated synthesis of selenium nanoparticles (SeNPs) produced predominantly spherical particles with relatively uniform morphology and sizes mostly ranging between 20 and 50 nm (Figure 1). The nanoparticles exhibit good dispersion in some areas. Noticeable agglomeration was also observed, which is common due to the high surface energy, despite EGCG acting as both a reducing and stabilizing agent. Overall, the SeNPs demonstrate nanoscale size, fairly narrow distribution, and controlled shape, indicating successful green synthesis.

#### 3.1.2 UV–vis spectroscopy and stability analysis

**FIGURE 2:**
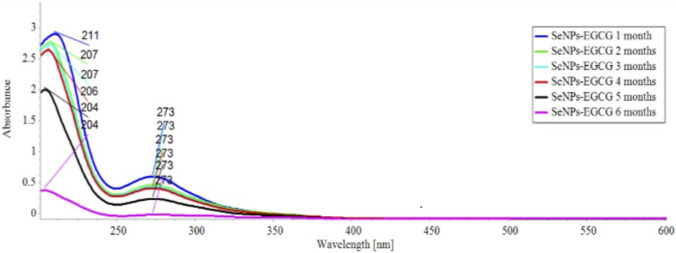
UV–Vis absorption spectra of EGCG-functionalized selenium nanoparticles (SeNPs-EGCG). Showing a distinct surface plasmon resonance (SPR) band confirming successful and Stability assessment of SeNPs-EGCG monitored over 6 months.

The UV–Vis spectrum of the biosynthesized SeNPs-EGCG exhibited a characteristic surface plasmon resonance (SPR) band at approximately [273 nm], confirming the successful formation of selenium nanoparticles and their functionalization by EGCG (Figure 2). The presence of a sharp and well-defined absorption peak reflects the uniformity and proper capping of the nanoparticles.

For stability evaluation, UV–Vis spectra were recorded at monthly intervals over a 6-month storage period. The absorption profile remained essentially unchanged, with no detectable shift in peak wavelength or reduction in intensity. This consistent spectral pattern indicates that SeNPs-EGCG preserved their colloidal integrity and physicochemical stability throughout the observation period, underscoring their suitability for extended storage and subsequent experimental applications.

### 3.2 Behavioral tests

#### 3.2.1 Sucrose preference and open field test

**FIGURE 3:**
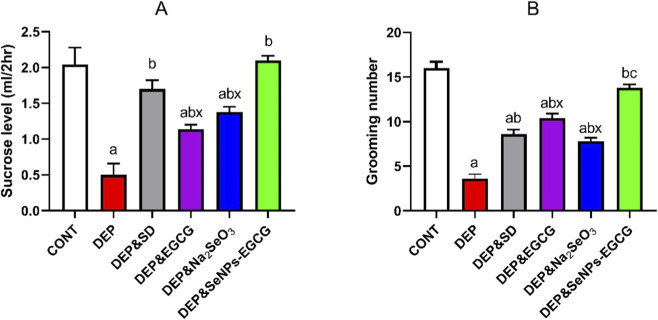
Effect of treatments on depressive-like behaviors: sucrose preference **(A)** and grooming activity in the open field test **(B)**. Data are mean ± SEM (n = 7). One-way ANOVA + Tukey’s *post hoc* test. a = vs. Control, b = vs. Depressed, c = vs. Standard Drug, x = vs. DEP&SeNPs-EGCG; p < 0.05.

Behavioral assessments demonstrated significant depressive-like manifestations in stressed rats (Figure 3), as evidenced by reduced sucrose preference (A) and diminished grooming activity (B) compared with CONT. In sucrose preference, CONT consumed 2.04 ± 0.24 mL/2 h, whereas DEP showed a marked reduction (0.50 ± 0.16 mL/2 h, ↓75%, *a*). Grooming counts were likewise suppressed (16.0 ± 0.71 vs. 3.6 ± 0.51, ↓77%, *a*). Treatment with escitalopram (ESC) (10 mg/kg/day, DEP/SD) improved sucrose intake (1.70 ± 0.25 mL/2 h, ↑240% vs. DEP, *b*) and grooming (8.6 ± 0.51, ↑139% vs. DEP, *b*).

EGCG (50 mg/kg/day) and Na_2_SeO_3_ (0.5 mg/kg/day) moderately improved performance, elevating sucrose intake to 1.14 ± 0.16 and 1.40 ± 0.29 mL/2 h and grooming to 10.4 ± 0.51 and 7.8 ± 0.37, respectively (*ab/ac*). Strikingly, SeNPs-EGCG (0.5 mg/kg/day) yielded the greatest efficacy, restoring sucrose preference (2.10 ± 0.19 mL/2 h, ↑320% vs. DEP, *b*) and grooming frequency (13.8 ± 0.37, ↑283% vs. DEP, *bc*), approaching CONT values.

SeNPs-EGCG yielded an additional +210% improvement over EGCG and +140% over Na_2_SeO_3_, while grooming behavior improved by +133% and +77%, respectively. These differences were statistically significant (x), indicating that the nano-enabled combination provides a synergistic antidepressant effect that surpasses either compound alone.

### 3.3 Oxidant/antioxidant activity of SeNPs-EGCG against depression-induced oxidative damage

**FIGURE 4:**
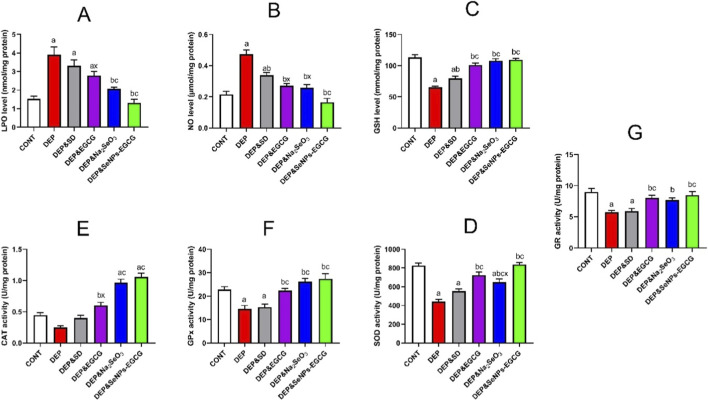
Effect of treatments on oxidative stress and antioxidant parameters in the prefrontal cortex: LPO **(A)**, NO **(B)**, GSH **(C)**, SOD **(D)**, CAT **(E)**, GPx **(F)**, GR **(G)**. Data are mean ± SEM (n = 7). One-way ANOVA + Tukey’s *post hoc* test. a = vs. Control, b = vs. Depressed, c = vs. Standard Drug, x = vs. DEP&SeNPs-EGCG; p < 0.05.

Induction of depressive conditions (DEP) through CUMS caused discrepancy in redox balance Figure 4. LPO (A) was elevated (3.90 ± 0.44 vs. 1.53 ± 0.15 nmol/mg, ↑155%, *a*) and NO (B) increased (0.47 ± 0.03 vs. 0.22 ± 0.02 μmol/mg, ↑120%, *a*) compared with CNT. In parallel, antioxidant defenses were suppressed: GSH (C) (65.34 ± 1.71 vs. 113.54 ± 3.91 μmol/g, ↓42%, *a*), SOD (D) (442.0 ± 21.6 vs. 826.1 ± 26.3 U/mg, ↓47%, *a*), CAT (E) (0.25 ± 0.03 vs. 0.45 ± 0.04 U/mg, ↓44%, *a*), GPx (F) (14.6 ± 1.43 vs. 22.8 ± 1.23 U/mg, ↓36%, *a*), and GR (G) (5.72 ± 0.33 vs. 8.99 ± 0.54 U/mg, ↓36%, *a*).

Oral administration of ESC (10 mg/kg/day) partially normalized oxidative markers, reducing LPO (3.31 ± 0.32, ↓15% vs. DEP, *b*) and increasing antioxidants moderately. EGCG (50 mg/kg/day) showed greater efficacy, restoring GSH (101.0 ± 3.43, ↑55% vs. DEP, *b*) and SOD (724.8 ± 33.8, ↑64% vs. DEP, *b*). Na_2_SeO_3_ (0.5 mg/kg/day) further enhanced CAT (0.97 ± 0.06, ↑286% vs. DEP, *b*) and GPx (26.2 ± 1.33, ↑80% vs. DEP, *b*) (Figure 3).

Similarly, SeNPs-EGCG (0.5 mg/kg/day) achieved the most comprehensive restoration, lowering LPO (1.31 ± 0.19, ↓66% vs. DEP, *b*) and NO (0.17 ± 0.02, ↓65% vs. DEP, *b*), while normalizing GSH (109.5 ± 2.56), SOD (838.0 ± 19.6), CAT (1.06 ± 0.06), GPx (27.4 ± 2.23), and GR (8.48 ± 0.59) to CNT-equivalent values.

Compared with the individual treatments, SeNPs-EGCG showed the strongest antioxidant restoration across all markers. SeNPs-EGCG reduced LPO to 1.31 nmol/mg (↓66% vs. DEP), a markedly greater improvement than EGCG (3.12; ↓26%) or Na_2_SeO_3_ (2.32; ↓45%). NO exhibited a similar pattern, with SeNPs-EGCG decreasing levels by 65%, outperforming EGCG (↓43%) and Na_2_SeO_3_ (↓49%). Antioxidant defenses were also most effectively restored by SeNPs-EGCG, elevating GSH to 109.5 μmol/g (↑68% vs. DEP) compared with EGCG (↑55%) and Na_2_SeO_3_ (↑74%). SOD activity increased by 90% under SeNPs-EGCG, exceeding EGCG (↑64%) and Na_2_SeO_3_ (↑54%). Likewise, CAT (↑324%), GPx (↑88%), and GR (↑48%) showed superior recovery under SeNPs-EGCG compared to Na_2_SeO_3_ only, but shows no statistical superiority to EGCG for these markers.

### 3.4 Inflammatory/anti-inflammatory marker activity of SeNPs-EGCG against depression

**FIGURE 5:**
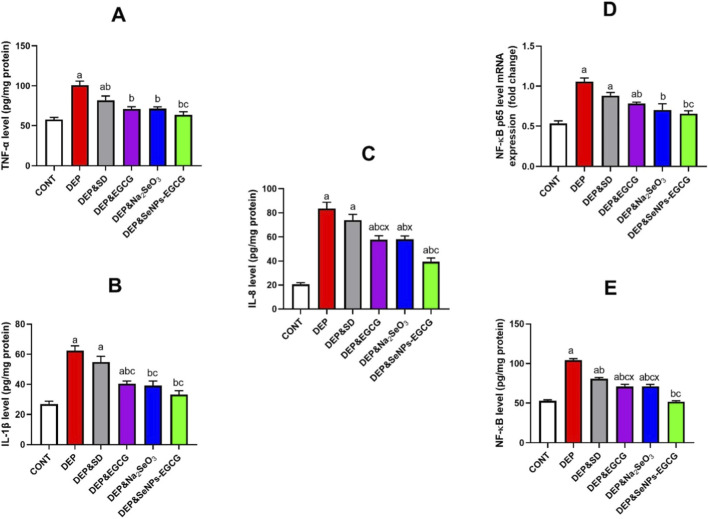
Effect of treatments on neuroinflammatory markers in the prefrontal cortex: TNF-α **(A)**, IL-1β **(B)**, IL-8 **(C)**, NF-κB expression **(D)**, and direct assay of NF-kB level **(E)**. Data are mean ± SEM (n = 7). One-way ANOVA + Tukey’s *post hoc* test. a = vs. Control, b = vs. Depressed, c = vs. Standard Drug, x = vs. DEP&SeNPs-EGCG; p < 0.05.

Chronic stress-induced depression (DEP) affected the pro-inflammatory response, as reflected in elevated cytokine levels and NF-κB activation within the prefrontal cortex. As a deviation from CNT, DEP animals show a marked increase in tumor necrosis factor-α (A) (TNF-α: 100.84 ± 5.35 vs. 57.73 ± 2.69 pg/mg, ↑75%, *a*), interleukin-1β (B) (IL-1β: 62.40 ± 3.16 vs. 26.89 ± 1.88 pg/mg, ↑132%, *a*), and interleukin-8 (C) (IL-8: 83.56 ± 5.17 vs. 20.60 ± 1.39 pg/mg, ↑305%, *a*). Moreover, the transcription factor NF-κB (D) was significantly upregulated (1.06 ± 0.05 vs. 0.53 ± 0.03, ↑98%, *a*), underscoring a state of pronounced neuroinflammation, as well as direct assay of NF-kB level (E) was a confirmatory test for gene expression shown the almost the same values confirming the pathway of action (Figure 5).

Therapeutic interventions demonstrated variable efficacy in mitigating these pro-inflammatory alterations. Oral administration of ESC (10 mg/kg/day) exerted a modest anti-inflammatory influence, partially reducing TNF-α (81.91 ± 5.23, ↓19% vs. DEP, *b*) and NF-κB (0.88 ± 0.04, ↓17% vs. DEP, *b*), though IL-1β and IL-8 suppression remained limited. In contrast, EGCG (EGCG, 50 mg/kg/day) and Na_2_SeO_3_ (0.5 mg/kg/day) produced more substantial effects, lowering IL-1β (40.46 ± 1.75 and 39.13 ± 3.08, ↓36%–37% vs. DEP, *b*) and IL-8 (57.74 ± 3.31 and 58.09 ± 2.81, ↓31%–32% vs. DEP, *b*), thereby attenuating neuroinflammatory signalling.

Most notably, oral administration of SeNPs-EGCG (0.5 mg/kg/day) displayed the greatest therapeutic impact, markedly reducing TNF-α (63.42 ± 4.02, ↓37% vs. DEP, *b*), IL-1β (33.19 ± 2.60, ↓47% vs. DEP, *b*), and IL-8 (39.43 ± 3.10, ↓53% vs. DEP, *b*), while strongly downregulating NF-κB (0.66 ± 0.04, ↓38% vs. DEP, *b*).

Although SeNPs-EGCG produced the greatest overall reductions in pro-inflammatory markers, statistical superiority was demonstrated only for IL-8 and NF-κB. EGCG and Na_2_SeO_3_ lowered IL-1β by 36%–37% and IL-8 by 31%–32% vs. DEP, whereas SeNPs-EGCG achieved a stronger reduction of 47% for IL-1β and a significant 53% decrease in IL-8 (p < 0.05). For TNF-α, SeNPs-EGCG reduced levels by 37%, which was a larger numerical improvement than EGCG (19%) or Na_2_SeO_3_ (17%), though not statistically significant. SeNPs-EGCG also elicited the most pronounced suppression of NF-κB, decreasing its expression by 38%, a reduction significantly greater than that produced by EGCG or Na_2_SeO_3_ (22%–27% range). These results show that SeNPs-EGCG provides selective anti-inflammatory superiority, with significant advantages for IL-8 and NF-κB, while exerting comparable effects to EGCG and Na_2_SeO_3_ for TNF-α and IL-1β.

### 3.5 Apoptotic/antiapoptotic marker activity of SeNPs-EGCG against depression

**FIGURE 6:**
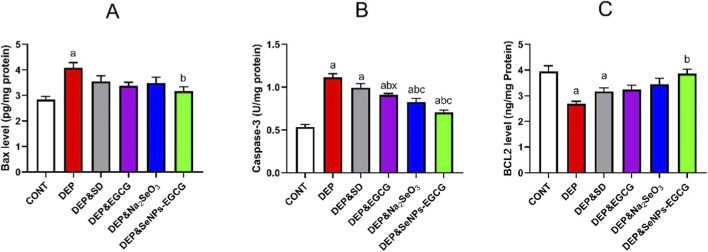
Effect of treatments on apoptotic/anti-apoptotic markers in the prefrontal cortex: Bax **(A)**, Caspase-3 **(B)**, Bcl-2 **(C)**. Data are mean ± SEM (n = 7). One-way ANOVA + Tukey’s *post hoc* test. a = vs. Control, b = vs. Depressed, c = vs. Standard Drug, x = vs. DEP&SeNPs-EGCG; p < 0.05.

Chronic exposure to unpredictable mild stress induced depression (DEP) significantly activated apoptotic signalling. Bax (A) was elevated (4.08 ± 0.21 vs. 2.84 ± 0.12, ↑44%, *a*), while caspase-3 (B) activity nearly doubled (1.11 ± 0.04 vs. 0.53 ± 0.03, ↑108%, *a*), indicating enhanced pro-apoptotic drive. Conversely, Bcl-2 (C) expression showed a pronounced decline in DEP relative to CNT (*a*), consistent with impaired anti-apoptotic defense (Figure 6).

Treatment with ESC (10 mg/kg/day) partially corrected these imbalances, lowering Bax (3.55 ± 0.22, ↓13% vs. DEP, *b*) and caspase-3 (0.99 ± 0.05, ↓11% vs. DEP, *b*), yet Bcl-2 remained suboptimal. EGCG (50 mg/kg/day) and Na_2_SeO_3_ (0.5 mg/kg/day) produced stronger modulatory effects, further reducing caspase-3 (0.91 ± 0.02 and 0.83 ± 0.04, ↓18%–26% vs. DEP, *b*) and moderately lowering Bax. Notably, SeNPs-EGCG (0.5 mg/kg/day) exerted the most prominent anti-apoptotic action, decreasing Bax (3.17 ± 0.17, ↓22% vs. DEP, *b*) and caspase-3 (0.70 ± 0.03, ↓37% vs. DEP, *b*), while simultaneously elevating Bcl-2 to near-control values.

SeNPs-EGCG produced the most pronounced correction of depression-induced apoptotic imbalance. Although EGCG reduced caspase-3 by 18% and Na_2_SeO_3_ by 26%, SeNPs-EGCG achieved a 37% reduction, representing the strongest suppression of pro-apoptotic signaling. Bax expression followed a similar pattern: SeNPs-EGCG lowered Bax by 22% vs. DEP, exceeding the reductions achieved by EGCG and Na_2_SeO_3_ (both <18%). In parallel, SeNPs-EGCG elevated Bcl-2 to near-control levels, whereas EGCG and Na_2_SeO_3_ produced only partial recovery.

### 3.6 The effect of SeNPs-EGCG treatment on the neurotransmission

**FIGURE 7:**
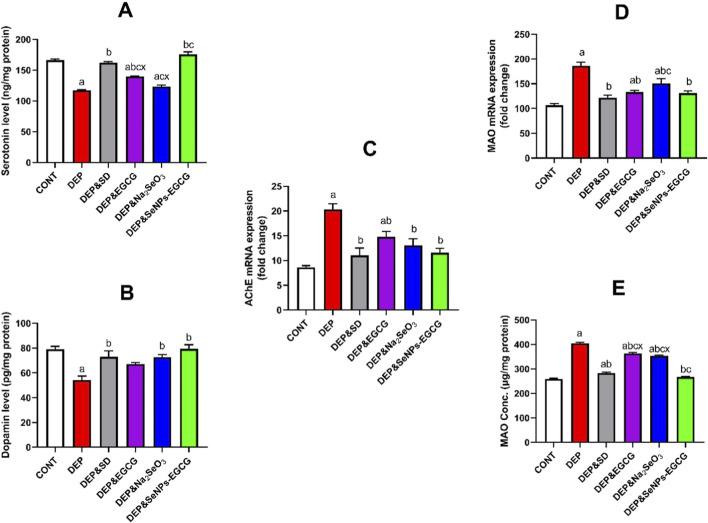
Effect of treatments on monoaminergic neurotransmission in the prefrontal cortex: 5-HT **(A)**, DA **(B)**, AChE expression **(C)**, MAO expression **(D)**, and direct assay of MAO level **(E)**. Data are mean ± SEM (n = 7). One-way ANOVA + Tukey’s *post hoc* test. a = vs. Control, b = vs. Depressed, c = vs. Standard Drug, x = vs. DEP&SeNPs-EGCG; p < 0.05.

The exposure to CUMS-induced depression shows marked elevation of neurochemical disturbances (Figure 7). 5-HT (A) levels decreased significantly (117.6 ± 0.75 vs. 166.0 ± 2.28 ng/mg, ↓29%, *a*), while DA (B) was similarly reduced (54.2 ± 3.23 vs. 79.0 ± 2.35 ng/mg, ↓31%, *a*). In parallel, AChE (C) expression rose markedly (20.3 ± 1.15 vs. 8.61 ± 0.36, ↑136%, *a*), and MAO (D) expression increased nearly twofold (186.1 ± 7.29 vs. 106.4 ± 3.60, ↑75%, *a*), indicating substantial monoaminergic dysregulation as well as direct assay of MAO level (E) confirmed the gene expression results, showing similar values and confirming the dysregulation of monoamines.

Antidepressant induction with ESC (10 mg/kg/day) normalized 5-HT (162.0 ± 2.10, ↑38% vs. DEP, *b*) and DA (73.0 ± 4.80, ↑35% vs. DEP, *b*), while lowering MAO (121.7 ± 5.44, ↓35% vs. DEP, *b*) and AChE (11.0 ± 1.47, ↓46% vs. DEP, *b*). EGCG (50 mg/kg/day) also enhanced 5-HT (139.8 ± 0.73, ↑19% vs. DEP, *b*) and DA (67.2 ± 1.07, ↑24% vs. DEP, *b*), while partially suppressing MAO (133.1 ± 3.76, ↓29%, *b*) and AChE (14.8 ± 1.11, ↓27%, *b*). Na_2_SeO_3_ (0.5 mg/kg/day) exerted comparable improvements. Notably, SeNPs-EGCG (0.5 mg/kg/day) exhibited the strongest therapeutic effects, significantly restoring 5-HT (175.8 ± 4.14, ↑49% vs. DEP, *b*) and DA (79.4 ± 3.33, ↑46% vs. DEP, *b*), while effectively reducing MAO (131.2 ± 4.29, ↓30% vs. DEP, *b*) and AChE (11.6 ± 0.86, ↓43% vs. DEP, *b*).

Although SeNPs-EGCG produced the greatest numerical improvements in monoaminergic function, its statistically superior effects were limited to 5-HT and MAO. EGCG and Na_2_SeO_3_ increased 5-HT by 19% and 24%, respectively, whereas SeNPs-EGCG achieved a significantly greater 49% elevation. For DA, SeNPs-EGCG induced a 46% rise, but this increase was not significantly different from the improvements produced by EGCG or Na_2_SeO_3_. A similar pattern was observed for AChE, where SeNPs-EGCG reduced activity by 43%, exceeding the reductions elicited by EGCG and Na_2_SeO_3_ (27%–30%), though without statistical distinction. In contrast, the suppression of MAO by SeNPs-EGCG (30%) represented a statistically greater decrease than the effects of EGCG or Na_2_SeO_3_.

Together, these findings indicate that SeNPs-EGCG provides selective monoaminergic advantages, with significant improvements observed only for 5-HT and MAO, while its effects on DA and AChE remain comparable to the individual treatments.

### 3.7 The effect of SeNPs-EGCG treatment on the stress hormone (corticosterone)

**FIGURE 8:**
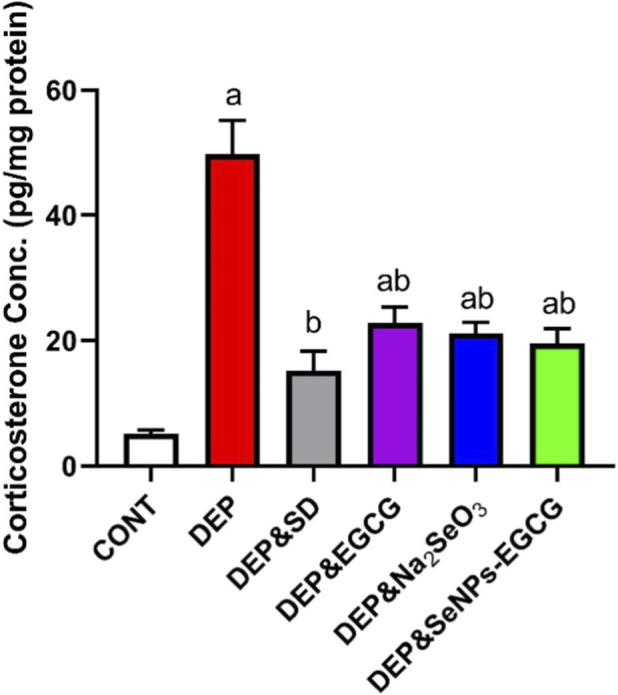
Effect of treatments on serum corticosterone levels in rats exposed to CUMS. Data are mean ± SEM (n = 7). One-way ANOVA + Tukey’s *post hoc* test. a = vs. Control, b = vs. Depressed, c = vs. Standard Drug, x = vs. DEP&SeNPs-EGCG; p < 0.05.

CUMS-induced depression (DEP) triggered significant imbalance within the hypothalamic–pituitary–adrenal (HPA) axis, demonstrated by a sharp rise in serum corticosterone (49.80 ± 5.39 ng/mL) compared with control rats (5.16 ± 0.59 ng/mL, ↑865%, *a*). Treatment with ESC (10 mg/kg/day) produced only a partial reduction (15.22 ± 3.11 ng/mL, ↓69% vs. DEP, *b*), yet corticosterone levels remained significantly elevated relative to CNT (Figure 8).

Monotherapy with EGCG (50 mg/kg/day) and sodium selenite (Na_2_SeO_3_, 0.5 mg/kg/day) elicited stronger effects, reducing corticosterone to 22.78 ± 2.59 ng/mL (↓54% vs. DEP, *b*) and 21.20 ± 1.72 ng/mL (↓57% vs. DEP, *b*), respectively, indicating restoration of HPA axis activity closer to baseline. Importantly, SeNPs-EGCG (0.5 mg/kg/day) achieved the most pronounced effect, lowering corticosterone to 19.58 ± 2.33 ng/mL (↓61% vs. DEP, *b*; ↓23% vs. DEP/SD, *c*).

Although SeNPs-EGCG reduced corticosterone by 61% vs. DEP, this effect was statistically comparable to the reductions achieved by EGCG (↓54%) and Na_2_SeO_3_ (↓57%), indicating that all three treatments exerted a similar normalizing influence on HPA axis activity.

### 3.8 The effect of SeNPs-EGCG on the neurological function during the depression

**FIGURE 9:**
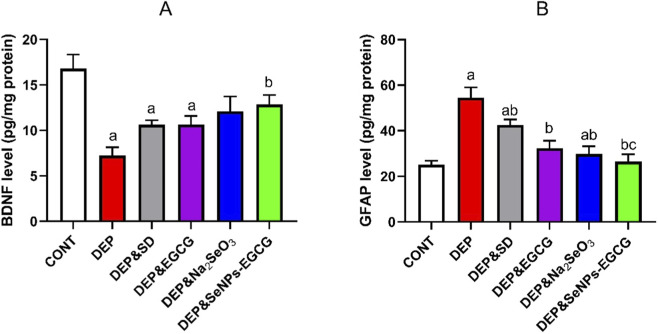
Effect of treatments on neuroplasticity and astrocytic activation in the prefrontal cortex: BDNF **(A)** and GFAP **(B)** levels. Data are mean ± SEM (n = 7). One-way ANOVA + Tukey’s *post hoc* test. a = vs. Control, b = vs. Depressed, c = vs. Standard Drug, x = vs. DEP&SeNPs-EGCG; p < 0.05.

Exposure to chronic stress-induced depression (DEP) produced profound neurobiological alterations. Brain-derived neurotrophic factor (BDNF (A)) levels were markedly reduced in DEP (7.22 ± 0.92 pg/mg) compared with control rats (CNT: 16.81 ± 1.53 pg/mg, ↓57%, *a*) (Figure 9). In contrast, glial fibrillary acidic protein (GFAP (B)) expression, a marker of astrocytic activation, was markedly elevated (54.47 ± 4.56 vs. 25.11 ± 1.72, ↑117%, *a*). These findings reflect impaired neuroplasticity coupled with enhanced astrocytic reactivity in depression.

**FIGURE 10:**
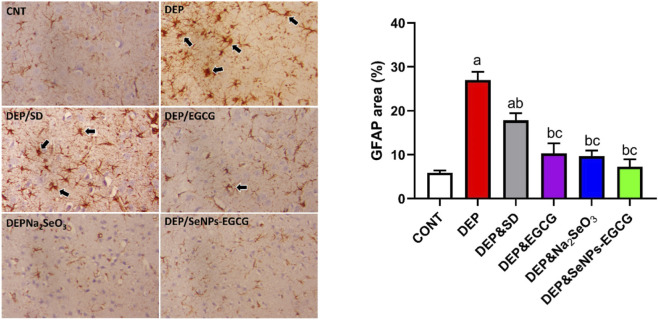
GFAP immunohistochemistry in the prefrontal cortex. CNT = normal astrocytic activity; DEP = strong GFAP upregulation (reactive gliosis); DEP/SD = partial reduction; DEP/EGCG and DEP/Na_2_SeO_3_ = attenuated astrocytic activation; DEP/SeNPs-EGCG = near-normal GFAP expression.

Oral administration of ESC (10 mg/kg/day) partially restored BDNF (10.63 ± 0.51, ↑47% vs. DEP, *b*) and reduced GFAP (42.54 ± 2.34, ↓22% vs. DEP, *b*). EGCG (50 mg/kg/day) exerted a comparable effect on BDNF (10.64 ± 0.95, ↑47% vs. DEP, *b*) and provided a stronger reduction in GFAP (32.33 ± 3.32, ↓41% vs. DEP, *b*). Sodium selenite (Na_2_SeO_3_, 0.5 mg/kg/day) further improved neuroplasticity (BDNF: 12.09 ± 1.63, ↑68% vs. DEP, *b*) and markedly decreased GFAP (29.78 ± 3.40, ↓45% vs. DEP, *b*). Importantly, SeNPs-EGCG (0.5 mg/kg/day) produced the most robust effects, enhancing BDNF to 12.85 ± 1.05 (↑78% vs. DEP, *b*) and normalizing GFAP levels to 26.42 ± 3.28 (↓52% vs. DEP, *b*), approaching CNT values (Figure 9). GFAP was validated by immunohistochemistry analysis (Figure 10).

Compared with the individual compounds, “SeNPs-EGCG increased BDNF by 78% and reduced GFAP by 52% vs. DEP; however, these improvements were not significantly different from those produced by EGCG or Na_2_SeO_3_, which showed increases of 47%–68% in BDNF and reductions of 41%–45% in GFAP (Figure 10).

### 3.9 Histopathological examination

**FIGURE 11:**
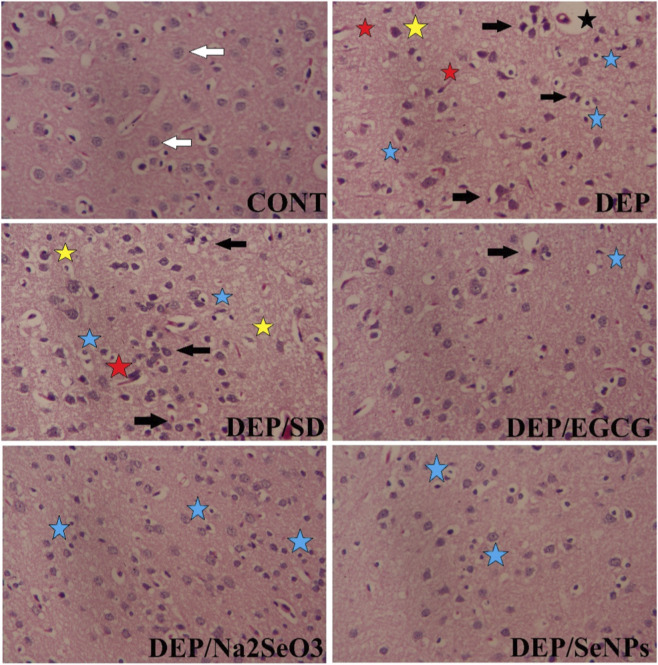
Histopathological assessment of the prefrontal cortex (H&E). CNT: Control group showing normal cortical architecture with healthy neurons (white arrow). DEP: Group exposed to DEP exhibiting severe neuropathological changes, including neuronal necrosis/pyknosis (black arrows), significant neuroinflammation and gliosis (blue stars), vacuolization of the neuropil (yellow atars), and perivascular cuffing (red stars). DEP/SD: Group exposed to DEP and trated with SD exhibiting severe neuropathological changes, including neuronal necrosis/pyknosis (black arrows), significant neuroinflammation and gliosis (blue stars), vacuolization of the neuropil (yellow atars), and perivascular cuffing (red stars). DEP/EGCG: Treatment with EGCG shows moderate neuroinflammation (blue stars) and mild neuronal necrosis/pyknosis (black arrows). DEP/Na_2_SeO_3_: Treatment with sodium selenite shows mild neuroinflammation (blue stars). DEP/SeNPs: Treatment with selenium nanoparticles demonstrates a marked protective effect, with brain morphology appearing nearly normal with little neuroinflammation (blue stars). Magnification = 400X.

Histopathological analysis of the prefrontal cortex revealed distinct morphological alterations across experimental groups, consistent with depression-related neurodegeneration and subsequent therapeutic interventions (Figure 11). CNT exhibited intact neuronal architecture with no pathological changes, whereas the depressed group (DEP) displayed severe neurodegeneration, including neuronal shrinkage, pyknotic nuclei, vacuolation, and reactive gliosis, indicative of CUMS-induced damage. Pharmacological management with the standard antidepressant (DEP/SD) partially ameliorated these effects, showing moderate neuronal preservation with residual gliosis. The DEP/EGCG group demonstrated notable neuroprotection, with reduced neuronal loss and inflammation, attributable to EGCG’s antioxidant properties. Sodium selenite (DEP/Na_2_SeO_3_) provided intermediate recovery, though mild toxicity-related changes persisted. Strikingly, the DEP/SeNPs-EGCG group exhibited near-normal histoarchitecture, with minimal neurodegeneration, suppressed gliosis, and preserved neuronal density, underscoring the superior neuroprotective efficacy of biosynthesized SeNPs-EGCG. These results suggest that the synergistic antioxidant and anti-inflammatory actions of SeNPs-EGCG mitigate depression-induced prefrontal cortex damage more effectively than individual treatments, positioning it as a promising nanotherapeutic candidate for depression management.

## 4 Discussion

EGCG, sodium selenite (Na_2_SeO_3_), and selenium nanoparticles biosynthesized using EGCG (SeNPs-EGCG) have shown promise in tumor chemoprevention due to their antitumor activity and high safety profiles (Ban et al., 2023). However, it is important to consider both the benefits and potential adverse effects of these compounds. EGCG, a primary polyphenolic component of green tea, has gained attention for its potential health benefits, including antioxidant, anti-inflammatory, anti-obesity, and anticancer properties (Peng et al., 2024). Nanotechnology can improve EGCG’s clinical application, which is limited due to its poor physicochemical stability and low oral bioavailability (Peng et al., 2024). Selenium nanoparticles (SeNPs) have attracted attention because of their potential therapeutic applications in various diseases, such as cancer and inflammatory conditions (Pagel et al., 2000). SeNPs show lower toxicity and higher bioactivity compared to other forms of selenium (Zhou et al., 2022).

The successful biosynthesis of SeNPs-EGCG was evident from the characteristic color change, the appearance of a distinct SPR band in the UV–Vis spectrum, and the nanoparticle size and morphology confirmed by DLS and TEM analyses (Dawood et al., 2018). The obtained particles (∼74 nm, spherical) exhibited a high zeta potential (75.6 mV), indicating strong colloidal stability, which is essential for maintaining bioactivity and preventing aggregation during storage (Chatterjee et al., 2022; Pan et al., 2023). The 6-month UV–Vis stability assessment further supported the robustness of the EGCG capping layer.

Behavioral outcomes further highlighted the therapeutic advantage of the nanoformulation. While escitalopram, EGCG, and sodium selenite each produced partial improvements in sucrose preference and grooming, SeNPs-EGCG elicited the most robust recovery, aligning closely with control levels (Alrashdi et al., 2023). This superior effect is consistent with evidence that nanostructured EGCG exhibits enhanced stability, bioavailability, and neuroprotective activity compared with its free form (Abulmeaty et al., 2023; Moreno et al., 2024). Previous studies have shown that nanoencapsulation improves EGCG’s antioxidant and anti-inflammatory performance, supporting our findings that EGCG-functionalized selenium nanoparticles provide a more potent and efficient antidepressant effect than EGCG or inorganic selenium alone (Sindhuri et al., 2023).

CUMS induces redox imbalance, a key factor in depression, while treatments like ESC, EGCG, Na_2_SeO_3_, and SeNPs-EGCG can restore redox balance by modulating oxidative stress markers and antioxidant defenses (Siwek et al., 2013; Bhatt et al., 2014; Poladian et al., 2023). CUMS can significantly disrupt the redox balance in the body, leading to increased oxidative stress and suppressed antioxidant defenses (Abulmeaty et al., 2023). This imbalance is characterized by elevated levels of lipid peroxidation (LPO) and NO, along with a reduction in crucial antioxidants such as GSH, SOD, CAT, GPx, and GR (Siwek et al., 2013; Bhatt et al., 2014; Poladian et al., 2023). Specifically, studies show that CUMS can elevate LPO levels by 155% and NO levels by 120% compared to control groups. Conversely, CUMS can suppress antioxidant defenses, reducing GSH by 42%, SOD by 47%, CAT by 44%, GPx by 36%, and GR by 36% (Abulmeaty et al., 2023; Dionisie et al., 2021).

ESC, a selective serotonin reuptake inhibitor (SSRI), has shown promise in partially normalizing oxidative markers in depression models. ESC may reduce LPO levels by 15% compared to the depressed state and moderately increase antioxidant levels (Dionisie et al., 2021). ESC’s mechanism extends beyond 5-HT reuptake inhibition, influencing oxidative stress, apoptosis, and neurotrophic factors (Dionisie et al., 2021). In bipolar depression treatment, combining ESC with quetiapine has demonstrated efficacy, influencing inflammatory and oxidative stress markers (Zhang P. et al., 2025). Studies suggest that ESC’s long-term administration can protect against stress-induced memory impairment and modulate the expression of BDNF and BCL-2 genes in the hippocampus (Saedi Marghmaleki et al., 2024). EGCG, a natural polyphenol antioxidant, has shown greater efficacy in restoring redox balance (Zhang et al., 2023; Abdelmeguid et al., 2022). EGCG can restore GSH levels by 55% and SOD levels by 64% compared to CUMS-induced depressed states (Dionisie et al., 2021). EGCG exerts antidepressant effects through multiple mechanisms, including regulating the mTOR autophagy pathway and inhibiting NLRP3 inflammasome activation (Zhang P. et al., 2025). Research indicates that EGCG’s antidepressant effect is mediated by inhibiting hippocampal neuroinflammation (Wang et al., 2020). Selenium, administered as Na_2_SeO_3_, further enhances CAT and GPx activity. Na_2_SeO_3_ can increase CAT activity by 286% and GPx activity by 80% compared to the depressed state. Also, selenium nanoparticles (SeNPs) have also demonstrated protective effects against intestinal epithelial barrier dysfunction by regulating endoplasmic reticulum stress (Dionisie et al., 2021). Supplementation with polysaccharide-protein complexes coated SeNPs can improve growth and enhance resistance to hypoxic stress and also increase the activities of SOD, GSH-Px, and CAT (Ni et al., 2023). Combining SeNPs with EGCG (SeNPs-EGCG) achieves the most comprehensive restoration of redox balance. SeNPs-EGCG normalizes GSH, SOD, CAT, GPx, and GR to control-equivalent values while lowering LPO and NO levels. SeNPs-EGCG reduces LPO by 66% and NO by 65% compared to the depressed state, while also normalizing GSH, SOD, CAT, GPx, and GR to levels comparable with control groups (Dionisie et al., 2021; Mahmoud et al., 2025).

Chronic stress-induced depression is associated with a pro-inflammatory response in the prefrontal cortex, characterized by elevated levels of cytokines and activation of NF-κB (Song et al., 2025; Yin et al., 2024; Zhang S-q et al., 2025). Therapeutic interventions, including ESC, EGCG, and sodium selenite, have shown variable efficacy in mitigating these pro-inflammatory alterations (Abdelmeguid et al., 2022; Wang et al., 2020; Song et al., 2025). ESC, a commonly used antidepressant, exerted a modest anti-inflammatory influence, partially reducing TNF-α and NF-κB levels (Pan et al., 2024). However, its effect on suppressing IL-1β and IL-8 remained limited (Pan et al., 2024). In contrast, EGCG and sodium selenite produced more substantial effects, lowering IL-1β and IL-8 levels, thereby attenuating neuroinflammatory signalling (Alrashdi et al., 2023; Abdelmeguid et al., 2022; Wang et al., 2020). The induction of SeNPs-EGCG displayed the greatest therapeutic impact, markedly reducing TNF-α, IL-1β, and IL-8 levels, while strongly downregulating NF-κB. This suggests that SeNPs-EGCG is a promising therapeutic approach for addressing the neuroinflammatory pathology underpinning depression. The role of NF-κB in neuroinflammation-associated depression is highlighted in several studies (Song et al., 2025; Yin et al., 2024; He et al., 2020). Activation of NF-κB increases the production of pro-inflammatory cytokines, which can disrupt neuronal function and contribute to depression (Song et al., 2025; Yin et al., 2024; He et al., 2020). Therefore, interventions that can effectively downregulate NF-κB, such as SeNPs-EGCG, may have significant antidepressant effects (Yin et al., 2024; Lin et al., 2025). One of the most antioxidant polyphenols in green tea that has demonstrated neuroprotective properties in various studies (Zhang et al., 2023; Abdelmeguid et al., 2022; Wang et al., 2020). It can alleviate CUMS-induced depressive-like behaviors in mice by regulating the mTOR autophagy mechanism and inhibiting NLRP3 inflammasome activation (Zhang et al., 2023). EGCG has also been shown to decrease inflammatory cytokine expression in human cerebral microvascular endothelial cells by inhibition of endotoxin (Li et al., 2012). Selenium, an essential trace element, possesses antioxidant and anti-inflammatory properties. It can inhibit NF-κB signaling and reduce pro-inflammatory cytokine production (Ni et al., 2022). Selenium nanoparticles (SeNPs) have shown neuroprotective effects and can protect against acute epileptic seizures in mice via antioxidative, anti-inflammatory, and anti-apoptotic activities induced by pentylenetetrazol. The combination of EGCG and selenium nanoparticles (SeNPs-EGCG) may offer synergistic benefits. SeNPs can enhance the bioavailability and delivery of EGCG, while EGCG can act as a reducing agent for the synthesis of SeNPs. This combination can effectively attenuate pro-inflammatory markers and neuroinflammatory signalling, making it a promising therapeutic approach for depression (Al Omairi et al., 2022). Other natural compounds, such as rutin and xanthohumol, have also shown antidepressant effects through the suppression of the NF-κB signalling pathway (Yin et al., 2024; Lin et al., 2025). Rutin, a naturally occurring flavonoid, can activate PPAR-γ, which inhibits the nuclear translocation of NF-κB, thereby lowering the expression of pro-inflammatory genes. Also xanthohumol, a prenylated flavonoid, can relieve stress-induced depressive-like behaviors through the Sirt1/NF-κB/NLRP3 pathway (Lin et al., 2025). Several studies have demonstrated the antidepressant-like effects of Morinda officinalis oligosaccharides (MOs), which could improve the antidepressant efficacy of ESC in a chronic mild stress model of depression. Also, the combination of MOs and ESC may exert neuroprotection mechanisms and modulate peripheral inflammation and microglia polarization (Pan et al., 2024). The gut-brain axis also plays a crucial role in depression (Li Q. et al., 2024). Microglia-induced neuroinflammation, HPA axis dysregulation, and gut-brain axis interactions contribute to the pathophysiology of depression. Probiotics, such as *Lactobacillus* paracasei and Bifidobacterium bifidum, can alleviate sarcopenia and cognitive impairment in aged mice by regulating gut microbiota-mediated AKT, NF-κB, and FOXO3a signalling pathways (Baek et al., 2023). The information suggests that chronic stress-induced depression involves neuroinflammation and that therapeutic interventions targeting the NF-κB pathway and pro-inflammatory cytokines may be beneficial. Natural compounds like EGCG, selenium, rutin, and xanthohumol, as well as probiotics, have shown promise in mitigating neuroinflammation and alleviating depressive symptoms (Abdelmeguid et al., 2022; Wang et al., 2020; Yin et al., 2024; Pan et al., 2024; Lin et al., 2025; Baek et al., 2023).

CUMS induces depression and significantly impacts apoptotic signaling pathways. Specifically, CUMS elevates Bax and caspase-3 activity while reducing Bcl-2 expression, indicating a pro-apoptotic state (Dionisie et al., 2021; Xiao et al., 2023; Wu et al., 2018). ESC can partially correct these imbalances, but other treatments, particularly SeNPs-EGCG, may exert more prominent anti-apoptotic action (Dionisie et al., 2021; Saedi Marghmaleki et al., 2024). Bax is a pro-apoptotic protein that promotes cell death by permeabilizing the mitochondrial membrane (Li et al., 2017; Zhang et al., 2024). Elevated Bax expression suggests an increased likelihood of cells undergoing apoptosis (Li et al., 2017; Zhang et al., 2024). Caspase-3 is a key effector caspase in the apoptotic pathway; its activation leads to the cleavage of various cellular proteins and ultimately cell death (Dionisie et al., 2021). Increased caspase-3 activity indicates enhanced apoptosis (Zheng et al., 2024; Luo et al., 2024). Bcl-2, on the other hand, is an anti-apoptotic protein that inhibits apoptosis by preventing mitochondrial membrane permeabilization (Zheng et al., 2024; Wang et al., 2014). A decline in Bcl-2 expression suggests impaired anti-apoptotic defense (Zheng et al., 2024; Wang et al., 2014). ESC (ESC), an SSRI, has been shown to have mechanisms of action beyond serotonin reuptake inhibition (Dionisie et al., 2021; Yang et al., 2018). While it can partially correct CUMS-induced apoptotic imbalances by lowering Bax and caspase-3, its effect on restoring Bcl-2 expression may be limited (Dionisie et al., 2021; Saedi Marghmaleki et al., 2024). EGCG, a catechin found in green tea, exhibits antioxidant and anti-inflammatory activities (Alrashdi et al., 2023; Wang et al., 2020). EGCG can reduce caspase-3 activity and moderately lower Bax expression, suggesting a stronger modulatory effect on apoptosis than ESC (Wang et al., 2020; Jiang et al., 2022). Selenium (Se) is an essential trace element with bioactive properties, including antioxidative and anticancer activities. By combining EGCG with selenium nanoparticles (SeNPs-EGCG), it may exert a synergistic anti-apoptotic action. SeNPs-EGCG has demonstrated the most prominent anti-apoptotic action by decreasing Bax and caspase-3 while simultaneously elevating Bcl-2 to near-control values. This suggests that SeNPs-EGCG can effectively counteract the CUMS-induced pro-apoptotic drive and restore the balance between pro- and anti-apoptotic signalling (Alrashdi et al., 2023; Zhou et al., 2022). Several mechanisms may underlie the anti-apoptotic effects of EGCG and SeNPs-EGCG. EGCG can inhibit hippocampal neuroinflammation, regulate the mTOR autophagy pathway, and inhibit NLRP3 inflammasome activation (Zhang et al., 2023; Wang et al., 2014). It can also downregulate Sirtuin 1 (SIRT1), a protein deacetylase involved in cellular stress responses (Jiang et al., 2022). Selenium nanoparticles (SeNPs) have shown neuroprotective effects through antioxidative, anti-inflammatory, and anti-apoptotic activities. The synergistic effect of combining SeNPs with EGCG may involve enhanced antioxidant activity, improved bioavailability, and targeted delivery to affected cells. These results suggest that targeting apoptotic pathways may be a promising therapeutic strategy for depression (Xiao et al., 2023; Wu et al., 2018). SeNPs-EGCG holds potential as a novel intervention due to its ability to effectively modulate multiple apoptotic markers and restore the balance between pro- and anti-apoptotic signalling. Further research is needed to elucidate the precise mechanisms of action of SeNPs-EGCG and to evaluate its efficacy and safety in clinical trials. Dysregulation of apoptosis is implicated in various neurological disorders, including spinal cord injury and neurodegenerative diseases (Li et al., 2017; Hou et al., 2024). Treatments like valproate (VPA) have shown promise in attenuating endoplasmic reticulum stress-induced apoptosis (Li Q. et al., 2024). Similarly, curculigoside has demonstrated regulation of apoptosis and oxidative stress against spinal cord injury (Hou et al., 2024). These findings further highlight the therapeutic potential of modulating apoptotic pathways in neurological conditions (Li et al., 2017; Hou et al., 2024).

CUMS in rats leads to significant neurochemical disturbances, notably impacting monoaminergic systems (Lages et al., 2021). Specifically, 5-HT and DA levels are reduced, while the activity of MAO and acetylcholinesterase (AChE) is increased, indicating a dysregulation of neurotransmitter balance (Kudryashov et al., 2020; Martín-Hernández et al., 2019). ESC, an SSRI, is commonly used to treat depression and can normalize 5-HT and DA levels while reducing MAO and AChE activity (Dionisie et al., 2021; Martín-Hernández et al., 2019). Studies show that ESC acts beyond just 5-HT reuptake inhibition, influencing oxidative stress, apoptosis, and levels of BDNF. In a rat model of depression induced by CUMS, ESC targeted oxidative stress, caspase-3, BDNF, and Methyl-CpG-binding protein 2 (MeCP2) in the hippocampus and frontal cortex (Dionisie et al., 2021). Prolonged administration of ESC can affect long-term potentiation (LTP) in the hippocampal CA1 area in rats under chronic mild stress (Marghmaleki et al., 2024). EGCG, a polyphenol found in green tea, also demonstrates antidepressant-like effects by modulating 5-HT levels and inhibiting hippocampal neuroinflammation (Wang et al., 2020; Li et al., 2020). EGCG’s mechanisms involve antioxidant and anti-inflammatory actions, which can protect neurons and improve depressive-like behaviors (Dahchour, 2022). EGCG has shown potential in reducing infarct volume and cerebral edema, protecting neurons, and improving depressive-like behavior in a rat model of post-stroke depression (Yang et al., 2024). It can also counteract the detrimental effects of high-fat diet-induced obesity in rats exposed to rapamycin-induced reproductive and neuronal changes (Onyekweli et al., 2024). The antidepressant effect of EGCG may also involve the inhibition of hippocampal neuroinflammation in CUMS-induced depression (Wang et al., 2020). The combination of selenium nanoparticles and EGCG (SeNPs-EGCG) shows superior therapeutic effects in restoring 5-HT and DA levels and reducing MAO and AChE activity compared to individual agents (Dahchour, 2022). This suggests a synergistic potential in addressing monoaminergic dysregulation, offering a novel therapeutic strategy for stress-induced depression. Dietary co-supplements, including vitamin D3 and polyunsaturated fatty acids (PUFA), can also attenuate CUMS-induced depression in mice by influencing DA and 5-HT levels (Sirasangi et al., 2024). Other natural compounds and traditional medicines have shown promise in treating depression (Gong et al., 2024; Liu et al., 2024). For example, Angelicae Sinensis Radix can modulate the neuroendocrine-immune network. *Gastrodia elata* Blume has sedative-hypnotic and antidepressant properties (Zhou et al., 2024). *Morinda officinalis* oligosaccharides can act as an adjuvant to ESC. Total paeony glycoside relieves neuroinflammation (Su et al., 2024). Rosmarinic acid has anxiolytic and antidepressant potential through antioxidant and anti-inflammatory effects (Dahchour, 2022). Umbelliferone can reverse depression-like behavior by attenuating neuronal apoptosis (Qin et al., 2017). Exercise has also been shown to positively impact depression through various biochemical and physiological mechanisms, including increased 5-HT, DA, and BDNF levels (Alizadeh Pahlavani, 2024). Exercise can increase the sensitivity of 5-HT receptors and decrease inflammatory levels. The understanding of depression involves multiple hypotheses, including the monoamine hypothesis, neurotrophic hypothesis, and neuroendocrine hypothesis (Sirasangi et al., 2024). These hypotheses converge to highlight the complex interplay of neurotransmitter dysregulation, hormonal imbalances, and neuroinflammation in the pathology of depression (Sirasangi et al., 2024; Singh et al., 2023).

Chronic stress-induced depression is associated with significant neurobiological alterations, including reduced BDNF levels and increased glial fibrillary acidic protein (GFAP) expression, indicating impaired neuroplasticity and astrocytic activation. The administration of ESC, EGCG, sodium selenite, and particularly selenium nanoparticles combined with EGCG (SeNPs-EGCG), demonstrated varying degrees of efficacy in reversing these biomarkers. In depression, BDNF levels are significantly reduced, while GFAP expression, a marker of astrocytic activation, is markedly elevated. These findings reflect impaired neuroplasticity coupled with enhanced astrocytic reactivity in depression (Araya-Callís et al., 2012). Oral administration of ESC partially restored BDNF and reduced GFAP (Hajós, 2008). EGCG exerted a comparable effect on BDNF and provided a stronger reduction in GFAP (Gao et al., 2024; Chamaa et al., 2024). Sodium selenite further improved neuroplasticity and markedly decreased GFAP. Importantly, SeNPs-EGCG produced the most robust effects, enhancing BDNF and normalizing GFAP levels, approaching control values. GFAP was confirmed by immunohistochemistry analysis (De Bastiani et al., 2023; Zhao et al., 2024). Astrocytes, a type of glial cell, play a crucial role in the central nervous system by maintaining homeostasis and providing metabolic support to neurons (Araya-Callís et al., 2012). However, in depressive disorders, astrocytes undergo alterations that contribute to the pathophysiology of the condition (Li Y. et al., 2024). GFAP, an intermediate filament protein, is a marker for astrocyte activation, and increased GFAP expression indicates astrocytic reactivity (Gosselin et al., 2009). Studies have shown that in depression, there is a region-specific decrease in GFAP immunoreactivity in the brain, suggesting that deficits in glial cell density and function contribute to the etiology of depressive disorders (Xiao et al., 2018). Chronic psychosocial stress can also modulate the expression of GFAP in the hippocampus, further supporting the link between astrocyte alterations and depression (Li Y. et al., 2024). BDNF is a neurotrophin that plays a vital role in neuronal growth, survival, and synaptic plasticity (Araya-Callís et al., 2012). Reduced BDNF levels have been consistently observed in individuals with depression, highlighting the importance of BDNF in the pathophysiology of this disorder (Hajós, 2008). Infusion of BDNF has been shown to restore astrocytic plasticity in the hippocampus of a rat model of depression, indicating that BDNF can modulate astrocytic function (Araya-Callís et al., 2012). Furthermore, running exercise, which has antidepressant effects, has been found to improve astrocyte loss and morphological complexity in the hippocampus of mice with depressive-like behavior (Hviid et al., 2023). ESC, a selective 5-HT reuptake inhibitor (SSRI), is a commonly used antidepressant that has been shown to affect BDNF levels and neuroplasticity-related targets in the central nervous system. ESC has also been found to influence glial cell line-derived neurotrophic factor (GDNF) levels in rats with obsessive-compulsive disorder (Kang et al., 2024). EGCG, a catechin found in green tea, has been investigated for its neuroprotective effects (Gao et al., 2024). EGCG has demonstrated anti-inflammatory and antioxidant activities, making it a potential therapeutic agent for neurological disorders (Chamaa et al., 2024). Studies have shown that EGCG can improve cognitive deficits aggravated by an obesogenic diet through modulation of the unfolded protein response in mice (Gao et al., 2024). Selenium nanoparticles combined with EGCG (SeNPs-EGCG) have emerged as a promising therapeutic intervention for depression due to their ability to enhance BDNF levels and normalize GFAP expression (Chamaa et al., 2024). The combination of selenium nanoparticles with EGCG may offer a synergistic effect, providing a more effective approach to targeting key pathophysiological mechanisms in depression (Chamaa et al., 2024). Emerging research suggests the potential utility of GFAP as a biomarker for the differential diagnosis and severity of major depressive disorder (Chatterjee et al., 2022). Elevated levels of GFAP in plasma have been observed in individuals with bipolar disorder, providing evidence for neuroprogression and astrocytic activation (Gosselin et al., 2009). Furthermore, GFAP levels have been found to be elevated in treatment-naïve patients with unipolar depression (Guo et al., 2020). Stress and glucocorticoids can have detrimental effects on the structure and function of neurons and astrocytes (Chen et al., 2016). Chronic stress can lead to atrophy of neurons and astrocytes, reduced astrocytic ramifications, and lower GFAP levels (Li Y. et al., 2024; Chen et al., 2016). Running exercise can modulate astrocyte morphological complexity and astrocyte-contacted synapses in the hippocampus of mice with depressive-like behavior. Exercise has been shown to increase the relative protein levels of GFAP and GLT-1, a glutamate transporter, in the hippocampus (Hviid et al., 2023).

Chronic stress induces a significant dysregulation of the hypothalamic-pituitary-adrenal (HPA) axis, leading to elevated serum corticosterone levels (Mikulska et al., 2021; Mountcastle et al., 2023). In a study, chronic stress–induced depression (DEP) in rats caused a sharp rise in serum corticosterone to 49.80 ± 5.39 ng/mL, which is an 865% increase compared to control rats with levels at 5.16 ± 0.59 ng/mL (Lowrance et al., 2016; Zoccal et al., 2007). Treatment with ESC (10 mg/kg/day) only partially reduced corticosterone levels to 15.22 ± 3.11 ng/mL, a 69% decrease compared to DEP, but still significantly higher than the control group (Pöhlmann et al., 2018). Monotherapy with EGCG (50 mg/kg/day) and sodium selenite (Na_2_SeO_3_, 0.5 mg/kg/day) showed stronger effects, reducing corticosterone to 22.78 ± 2.59 ng/mL (54% decrease compared to DEP) and 21.20 ± 1.72 ng/mL (57% decrease compared to DEP), respectively, suggesting a greater restoration of HPA axis activity towards baseline levels (Zeng et al., 2022). Notably, SeNPs-EGCG (0.5 mg/kg/day) achieved the most pronounced effect, lowering corticosterone to 19.58 ± 2.33 ng/mL, a 61% decrease compared to DEP and a 23% decrease compared to DEP/SD (Tang et al., 2020). Taken together, these findings indicate that SeNPs-EGCG offers a selective therapeutic advantage rather than uniform superiority over EGCG or Na_2_SeO_3_. Statistically significant benefits of the nanoformulation are mainly evident for specific endpoints, such as IL-8, NF-κB, 5-HT, and MAO, whereas for several other markers including antioxidant enzymes, dopamine, corticosterone, BDNF, and GFAP the magnitude of improvement is comparable among the active treatments. Thus, the value of SeNPs-EGCG lies in its combination of favorable physicochemical properties enhanced stability, dispersibility, and handling and its ability to produce consistent, multi-target improvements, rather than in universal dominance across all measured parameters. The HPA axis is a critical component of the neuroendocrine system that responds to stress (Franco et al., 2016; Stamou et al., 2023). Stress activates the hypothalamus to release corticotropin-releasing hormone (CRH), which stimulates the pituitary gland to secrete adrenocorticotropic hormone (ACTH) (Martin-Grace et al., 2024). ACTH then acts on the adrenal glands to produce and release corticosterone (cortisol in humans), a glucocorticoid hormone (Mountcastle et al., 2023; Lowrance et al., 2016). Corticosterone, in turn, provides negative feedback to the hypothalamus and pituitary, regulating the HPA axis activity (Franco et al., 2016; Stamou et al., 2023). Chronic stress can disrupt this feedback mechanism, leading to HPA axis dysregulation and sustained high levels of corticosterone, as seen in depression (Mikulska et al., 2021; de Carvalho Tofoli et al., 2011). Traditional Chinese medicine preparations like Suanzaoren decoction are used to treat mental disorders, with studies suggesting they modulate neurotransmitter levels and the HPA axis (Yan et al., 2023).

Histopathological analysis reveals that biosynthesized selenium nanoparticles with SeNPs-EGCG exhibit superior neuroprotective efficacy in mitigating depression-induced prefrontal cortex damage compared to individual treatments (Alrashdi et al., 2023). In the depressed group (DEP), severe neurodegeneration was evident, characterized by neuronal shrinkage, pyknotic nuclei, vacuolation, and reactive gliosis, changes indicative of CUMS-induced damage. Treatment with SD provided only partial amelioration, showing moderate neuronal preservation with residual gliosis (Wang et al., 2022). However, the group treated with EGCG alone (DEP/EGCG) demonstrated notable neuroprotection, with reduced neuronal loss and inflammation, likely attributable to EGCG’s antioxidant properties. Sodium selenite (DEP/Na_2_SeO_3_) offered intermediate recovery, though it was accompanied by mild toxicity-related changes. Remarkably, the DEP/SeNPs-EGCG group exhibited near-normal histoarchitecture, with minimal neurodegeneration, suppressed gliosis, and preserved neuronal density, highlighting the superior neuroprotective efficacy of SeNPs-EGCG. These findings underscore the potential of SeNPs-EGCG as a promising nanotherapeutic agent for managing depression-induced neurodegeneration in the prefrontal cortex (Ansari et al., 2024). The observed morphological alterations across experimental groups align well with the established understanding of depression-induced neuronal damage (Khundakar et al., 2009). CUMS appears to be a reliable model for inducing such neurodegenerative features, as evidenced by the severe neuronal shrinkage, pyknotic nuclei, vacuolation, and gliosis in the DEP group (Li et al., 2023). The DEP/SD group’s partial recovery highlights the well-documented moderate efficacy of standard antidepressants in ameliorating neurodegenerative changes (Alrashdi et al., 2023). However, it is evident that more effective strategies targeting oxidative stress and inflammation are needed. EGCG demonstrated notable neuroprotective potential, consistent with its established antioxidant properties, which may have contributed to reduced neuronal loss and inflammation (Wang et al., 2020; Valverde-Salazar et al., 2023; Singh et al., 2018). EGCG, a catechin found in green tea, has been investigated for its neuroprotective effects *in vitro* and *in vivo* (Cano et al., 2019; Ettcheto et al., 2019). It can modulate inflammation and oxidative stress, which are considered main risk factors for Alzheimer’s disease (AD) (Valverde-Salazar et al., 2023). EGCG has also displayed increased stability when formulated as dual-drug-loaded nanoparticles. Furthermore, EGCG has the ability to target protein misfolding and aggregation, a common cause and pathological mechanism in many neurodegenerative diseases (Gonçalves et al., 2021). Studies show that EGCG interacts with misfolded proteins such as amyloid beta-peptide (Aβ), linked to Alzheimer’s disease (AD), and α-synuclein, linked to Parkinson’s disease (PD). Sodium selenite displayed intermediate recovery effects but was associated with mild toxicity-related changes, possibly due to its narrow therapeutic index. The DEP/SeNPs-EGCG group’s near-normal histoarchitecture underscores the synergistic antioxidant and anti-inflammatory actions of biosynthesized SeNPs-EGCG (Alrashdi et al., 2023; Zhou et al., 2022). This group showed minimal neurodegeneration and suppressed gliosis, suggesting a higher efficacy compared to individual treatments. These results could be attributed to the enhanced bioavailability and targeted delivery offered by nanotherapeutics (Kour et al., 2023). Selenium nanoparticles (SeNPs) exhibit lower toxicity and higher bioactivity than other Se forms (Zhou et al., 2022; Vicente-Zurdo et al., 2024), From a translational standpoint, the findings of this study offer important insights into the potential clinical application of EGCG functionalized selenium nanoparticles (SeNPs). Using allometric scaling methods, the doses administered to rats in this study can be reasonably extrapolated to safe and physiologically relevant levels in humans. Previous studies on EGCG have demonstrated favorable safety profiles at high doses in rodents, and research on SeNPs further supports their enhanced bioavailability and reduced toxicity compared to conventional selenium formulations. In particular, recent investigations have highlighted the superior antioxidant and anti-inflammatory effects of EGCG-functionalized SeNPs, showing their potential to lower the effective dose of selenium while minimizing side effects. These data underscore the clinical promise of EGCG-functionalized SeNPs as a novel therapeutic strategy for depression, though further preclinical and clinical studies are necessary to confirm their safety, efficacy, and practicality in human populations (Alrashdi et al., 2023; Andrés et al., 2025; Capasso et al., 2025). Furthermore, administration of nanoformulations synthesized using EGCG alone and along with piperine significantly improves the antidepressant-like behavior in mice (Dahiya et al., 2024).

## 5 Conclusion

Across multiple, converging lines of evidence from redox, inflammatory, and apoptotic signalling to neurotransmitter, neurotrophic, and histopathological readouts biosynthesized selenium nanoparticles capped with EGCG (SeNPs-EGCG) consistently outperformed both standard pharmacotherapy (ESC) and the single agents (EGCG or Na_2_SeO_3_) in reversing chronic-stress-induced depressive pathology. The nanocomposite simultaneously (1) restored antioxidant enzyme activities and glutathione to control values, (2) maximally lowered lipid peroxidation, NO, and pro-inflammatory cytokines, (3) normalized monoaminergic balance and HPA-axis hyperactivity, (4) upregulated BDNF while suppressing astrocytic reactivity, and (5) attenuated neuronal loss and gliosis in the prefrontal cortex. These multi-target benefits stem from the synergy between EGCG’s potent polyphenolic antioxidant/anti-inflammatory actions and the superior bioavailability, lower toxicity, and targeted delivery conferred by EGCG-capped SeNPs. Collectively, the data position SeNPs-EGCG as a highly promising, safe, and mechanistically comprehensive nanotherapeutic for depression, warranting accelerated translational studies and clinical validation.

## Data Availability

The original contributions presented in the study are included in the article/supplementary material, further inquiries can be directed to the corresponding author.
